# Common Dolphin (*Delphinus delphis*) Bycatch in New Zealand Commercial Trawl Fisheries

**DOI:** 10.1371/journal.pone.0064438

**Published:** 2013-05-22

**Authors:** Finlay N. Thompson, Edward R. Abraham, Katrin Berkenbusch

**Affiliations:** Dragonfly Science, Wellington, New Zealand; University of Canterbury, New Zealand

## Abstract

Marine mammals are regularly reported as bycatch in commercial and artisanal fisheries, but data are often insufficient to allow assessment of these incidental mortalities. Observer coverage of the mackerel trawl fishery in New Zealand waters between 1995 and 2011 allowed evaluation of common dolphin *Delphinus delphis* bycatch on the North Island west coast, where this species is the most frequently caught cetacean. Observer data were used to develop a statistical model to estimate total captures and explore covariates related to captures. A two-stage Bayesian hurdle model was used, with a logistic generalised linear model predicting whether any common dolphin captures occurred on a given tow of the net, and a zero-truncated Poisson distribution to estimate the number of dolphin captures, given that there was a capture event. Over the 16-year study period, there were 119 common dolphin captures reported on 4299 observed tows. Capture events frequently involved more than one individual, with a maximum of nine common dolphin observed caught in a single tow. There was a peak of 141 estimated common dolphin captures (95% c.i.: 56 to 276; 6.27 captures per 100 tows) in 2002–03, following the marked expansion in annual effort in this fishery to over 2000 tows. Subsequently, the number of captures fluctuated although fishing effort remained relatively high. Of the observed capture events, 60% were during trawls where the top of the net (headline) was <40 m below the surface, and the model determined that this covariate best explained common dolphin captures. Increasing headline depth by 21 m would halve the probability of a dolphin capture event on a tow. While lack of abundance data prevents assessment of the impact of these mortalities on the local common dolphin population, a clear recommendation from this study is the increasing of headline depth to reduce common dolphin captures.

## Introduction

Direct interactions between fisheries and marine mammals frequently occur when fishing operations overlap with the distribution of pinniped and cetacean populations [1]–[3]. In the majority of incidents, entanglement and entrapment in fishing gear result in injury and mortality, and incidental captures of marine mammals have been documented for a variety of fisheries worldwide [4]–[6]. For some marine mammal populations and species, these incidental captures pose a serious threat [7]–[9]. Cetaceans are particularly vulnerable to increased mortalities as they have slow life histories and limited potential for population increase [10].

A number of cetacean species are attracted to fishing vessels, and have been observed to feed in association with trawlers [11], [12]. One species that features prominently in bycatch reports across different fisheries and regions is short-beaked common dolphin *Delphinus delphis* Linnaeus, 1758 (hereafter referred to as “common dolphin”) [13]–[16]. This species is globally distributed in temperate, subtropical and tropical regions, where it is often abundant in coastal waters [17]. Common dolphin often form large aggregations (up to several thousand individuals), including multi-species associations with other cetaceans, such as pilot whale *Globicephala* sp. and striped dolphin *Stenella coeruleoalba* [18]–[20]. Based on its global distribution and abundance, the conservation status of common dolphin is “least concern” [21]. On a smaller spatial scale, however, there are several separate populations that are in serious decline, such as in the Mediterranean and Black seas [22]. Furthermore, distinct morphological differences indicate the existence of potential sub-species in some regions, including the Black Sea [21].

In New Zealand waters, common dolphin are present in coastal areas throughout the country, with a number of recent studies focused on northern populations [23]–[26]. Although there are no population estimates for this region, they are considered to be abundant, often forming schools of over a hundred individuals [20]. As has been documented for populations elsewhere [27], common dolphin in New Zealand show differences in their distribution and habitat use [20]. While small groups of common dolphin are present year-round in shallow waters (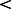
20 m depth) in some regions [20], they generally exhibit seasonal inshore/offshore movement, residing in coastal waters during spring and summer, before moving further offshore during autumn [20]. Their inshore/offshore migration also occurs on a diel basis, which seems to correspond with the exploitation of different food sources and the movement of prey [25].

Common dolphin feed predominantly on epipelagic and pelagic species, such as schooling fishes and squids [29]–[31]. In New Zealand waters, the diet of common dolphin largely consists of jack mackerel (*Trachurus* spp.), anchovy (*Engraulis australis*), and arrow squid (*Nototodarus* spp.) [25]. Their prey preference makes common dolphin susceptible to trawl fisheries targeting the same species [32]. The incidental capture of this species has been documented for pelagic trawl fisheries across geographical regions, including different parts of the Atlantic Ocean [32], [33], the North Sea, Mediterranean Sea, and Pacific Ocean (see review in [5]). The majority of records, however, are anecdotal or insufficient to allow a systematic assessment of bycatch numbers and potential impacts for this (and other) marine mammal species (but see for example, [32]).

In view of the scarcity of quantitative assessments, the present study was aimed at estimating the number of common dolphin captured in commercial trawl fisheries in New Zealand waters. A considerable proportion of common dolphin are caught and killed by vessels targeting mackerel, including jack mackerel (*Trachurus declivis*, *T. murphyi*, and *T. novaezelandiae*) or blue mackerel (*Scomber australasicus*) [26]. This mackerel fishery is concentrated on the west coast of New Zealand's North Island, and had sufficient observer coverage to allow the development of a statistical model for the estimation of total captures (including unobserved fishing effort) based on observed trawls (or tows) and capture records. In addition to obtaining total bycatch estimates, the modelling approach also allowed the identification of factors that were associated with common dolphin captures.

## Methods

### Data sources

The statistical model built to estimate common dolphin captures was based on fishing-effort and observer data for the 16-year period between 1 October 1995 and 30 September 2011 (the fishing year in New Zealand runs from 1 October to 30 September, and the data analysis and presentation followed this format). Fishing data were obtained from records of trawler activity reported by commercial fishers on effort return forms, including the date and time of trawl effort, the position of the start and end of each trawl, the target species, catch weight, and details of the fishing gear used. Incidental captures of protected species were recorded by government observers on-board commercial fishing vessels within New Zealand's Exclusive Economic Zone (EEZ). Observer data included the identity of the species captured, and the time and location of the captures and of every observed trawl.

Both fishing effort and observer records were prepared and linked, correcting for errors in date, time, and position fields. Observer records were linked to the fisher-reported effort data by comparing the start and end times, location, and target species for each vessel. To accurately predict captures on the unobserved trawls, it was necessary to develop the model with factors that were available on the trawls that were not observed. This requirement limited available data to those recorded by the fishers.

**Figure 1:**
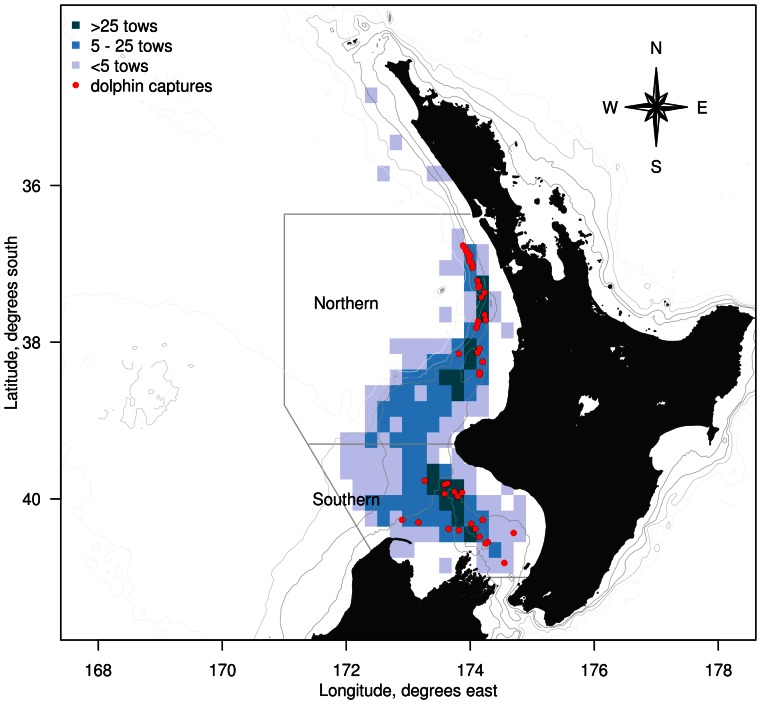
Commercial trawl effort in the west coast North Island region, New Zealand. Mean annual trawl effort (number of tows) of the commercial mackerel (*Trachurus* spp. and *Scomber australasicus*) fishery between 1 October 1995 and 30 September 2011, including locations of observed common dolphin (*Delphinus delphis*) captures. Also indicated are the boundaries of the area modelled for estimating common dolphin captures.

An area on the west coast of the North Island was used to select data for modelling and analysis, as it included the mackerel fishery region where common dolphin captures have been observed. This area was enclosed by a line extending north along longitude 173° 2.8′ E, a line across Cook Strait (between 3 North and South islands) at latitude 41° S, boundary at 171° E, and the boundary of New Zealand's EEZ (Figure 1). To provide higher spatial resolution, the area was divided into northern and southern sub-areas at latitude 39°18′ S.

### Common dolphin capture model

The large number of tows without captures in the observer data resulted in an unbalanced data set owing to a disproportional high number of zeros. Three different models structures were initially trialled to account for this high number of zeros, including a negative-binomial model with overdispersion, a zero-inflated Poisson model which allows extra zeros, and a hurdle model which included a two-stage Bayesian model that separately predicts the probability of capture events occurring and the number of captures on each capture event. The latter model was chosen, as it was difficult to estimate the overdispersion parameter in the negative-binomial model, and the likelihood that an observer failed to report the capture events (alongside the likelihood of captures occurring) in the zero-inflated model structure. The difficulties in estimating these parameters increases the uncertainty in the resulting estimates, so that the two-stage Bayesian model was the preferred model structure used here. Models of this kind are called hurdle models [34], [35], and are appropriate when different processes are influencing the occurrence of captures and the number of animals caught in each capture event.

In the first stage, a logistic generalised linear model estimated the probability of capturing common dolphin on a given tow as a linear function of a number of covariates. Given that there was a capture event, the number of captures was then estimated in the second stage by sampling from a zero-truncated Poisson distribution. In addition to estimating total captures, the model determined the relationship between covariates and common dolphin captures.

The statistical model estimated the probability, 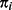, of capturing dolphins on a tow, 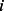. A year effect, 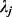 was estimated for each year, 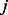, allowing for annual variation in the capture event rates that was unrelated to the covariates, 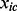. The contribution of each covariate, indexed by 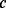, was governed by a regression coefficient, 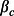, that was estimated by the model. The logit transform of the capture event probability was defined as the sum of the year effect, 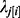, and the covariates:
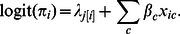
(1)Diffuse normal priors were given to the regression coefficients, 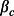, and to the mean of the year effects, 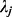. A half-Cauchy prior, with a scale of 25, was given to the variance of the year effects. Uninformative priors were given to all the parameters and hyper-parameters (following recommendations from [36]).

On tows where common dolphin captures occurred, the captures were assumed to follow a zero-truncated Poisson distribution with size 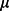. The use of a zero-truncated distribution reflected the structure of the hurdle model (if a capture event occurred the number of dolphins caught must have been one or more). The probability that 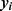 dolphins were captured on tow 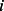 was given by
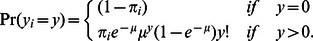
The size, 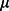, was given a prior that was uniform between 0.5 and 30. It would be possible for the size of the truncated Poisson distribution, 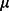, to vary with the value of covariates on each tow. However, an initial exploration suggested that there was no consistent variation of the size 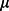 with any available covariates.

Estimates were prepared for groups of tows, grouped by fishing year, 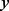, and vessel, 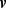. The estimated total number of dolphins captured in a group, 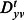, was calculated as the sum of actual reported captures on observed tows, 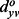, and estimated captures on the unobserved tows, 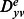,
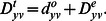
(2)Total captures in a year were obtained by summing the captures over all vessels fishing in that year, 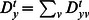.

The model was coded in the BUGS language [37], a domain-specific language for describing Bayesian models (please see Appendix S1 for the BUGS code used). The model was fitted with the software package JAGS [38], using Markov chain Monte Carlo (MCMC) methods. To ensure that the model had converged, a burn-in of 10 000 iterations was made. The model was subsequently run for another 100 000 iterations and every 20th iteration was kept. Two chains were fitted to the model, and the output included 5000 samples of the posterior distribution from each chain. Model convergence was assessed using diagnostics provided by the CODA package for the R statistical system [39]. To test whether the model produced a suitable representation of the data, simulations of observed captures were made using randomly chosen samples from the Markov chains and visually compared with the observed captures [40]. A comparison was made of the frequency distribution of the number of dolphins caught during capture events, between the observed data and predictions from samples from the Markov chains.

### Covariate selection

The model structure allowed for the dolphin capture event probability to depend on covariates. A step analysis was used to identify the covariates that had explanatory power [41]. Maximum likelihood methods were used to fit a binomial generalised linear model to the observed capture events, trying different combinations of factors (see list of potential covariates in Table 1). At each stage of the analysis, the model was fitted repeatedly, with each of the covariates included (or removed) in turn and selection of the covariate that produced the greatest reduction in the Akaike Information Criterion [42]. Steps continued until the deviance was not reduced by more than 1%. Placing a requirement on the deviance reduction prevented the inclusion of covariates that had little explanatory power. Catch weight, tow duration, night hours, bottom depth, and fishing depth, were all included both directly and as a log-transform (with one tonne and one hour added to catch weight and night hours, respectively, before performing the transformation).

**Table 1:** Potential covariates considered for inclusion in the model.

Covariate	Description
Trawl speed	Fishing speed (knots) from the Trawl Catch Effort Processing Return (TCEPR) data.
Trawl duration	The duration of tows (hours) from start and end times recorded on TCEPR forms.
Fishing depth	The depth of the net ground line (metres).
Headline height	The height of the net opening (metres).
Headline depth	The depth of the top of the net (metres), derived by subtracting the headline height from the ground line depth (both recorded on TCEPR forms). Indicates the depth of the top of the net.
Bottom depth	Minimum depth at either the start or end positions of tows (metres), derived using ETOPO2v2 bathymetric data [55], [56].
Depth factor	Bottom depth as a factor, with tows in water less than 210 m defined as shallow, and other tows defined as deep.
Catch weight	Total catch weight of each trawl (tonnes) as recorded on the TCEPR forms.
Sub-area	The west coast North Island region divided into two sub-areas (north and south of 39°18′ S) and each included as a factor variable.
Light condition	A three-valued factor that classified tows according to the time of the haul and the phase of the moon. The three levels were set following initial exploration and were: light (net hauled between dawn and dusk, or between dusk and midnight on a moonlit night), dark (net hauled between dusk and midnight on a dark night, or between midnight and dawn on a moonlit night), and black (net hauled between midnight and dawn on a dark night). The illumination of the moon and time of dawn and dusk were calculated using standard algorithms [57]. The night was classified as moonlit if more than 17% of the moon's disc was illuminated. Dawn and dusk were defined as when the centre of the sun's disk was 6° below the horizon (civil dawn and dusk).
Moon illumination	Fractional illumination (percentage) of the moon's disc, calculated using standard algorithms [57].
Night hours	The number of night hours during a trawl, calculated as the number of hours of the tow between civil dawn and dusk.
Month	Months of the year as a factor variable.
Season	A grouping of months into quarters (January to March, April to June, July to September, and October to December), included as a factor variable.
Nation	Factor indicating which flag each vessel was flying: Russia, New Zealand, Japan, Korea, or FOC (a flag of convenience).

Definition of variables that were included in the step analysis to select covariates for estimating common dolphin *Delphinus delphis* bycatch in a commercial trawl fishery in New Zealand waters.

## Results

### Observed captures

Between 1995–96 and 2010–11, there were 135 observed common dolphin captures in commercial trawl fisheries in New Zealand waters. The majority of observed captures (119) were in the mackerel fishery operating on North Island's west coast. All 119 observed captures occurred on vessels that were longer than 90 m. As a consequence, estimates of common dolphin captures were based on observer data from vessels over 90 m length that targeted jack mackerel or blue mackerel on at least one tow per fishing trip. These trips were defined as the large-vessel mackerel fishery. Other observed marine mammal captures in this fishery involved 11 pilot whales *Globicephala* spp. and 16 New Zealand fur seal *Arctocephalus forsteri*.

There was a total of 23 499 tows reported by the large-vessel mackerel fishery over the 16-year study period (Table 2). Over this time, trawl effort was initially low, but increased substantially between 1999–00 and 2002–03 (Table 2). Since then, fishing effort has generally been around 2000 tows per year, with a decrease in trawl effort in the most recent fishing year (2010–11), when 1551 tows were fished. Observer coverage between 1995–96 and 2010–11 fluctuated considerably, between 7 and 70%, with at least 20% of all tows observed in most fishing years.

**Table 2:** Annual summary of common dolphin *Delphinus delphis* captures in the west coast North Island region.

Year	Effort	% obs.	Cap.	Events	Rate	Est. captures		Est. capture rate	
	Tows		Dolphins	Tows	per 100 tows	Mean	95% c.i.	Mean	95% c.i.
1995–96	406	29.6	2	1	1.67	5	2–16	1.20	0.49–3.94
1996–97	230	70.4	0	0	0.00	0	0–4	0.15	0.00–1.74
1997–98	560	38.9	0	0	0.00	2	0–9	0.30	0.00–1.61
1998–99	350	24.0	0	0	0.00	3	0–15	1.00	0.00–4.29
1999–00	412	17.2	1	1	1.41	8	1–27	1.83	0.24–6.55
2000–01	974	12.2	1	1	0.84	12	1–40	1.28	0.10–4.11
2001–02	1577	7.0	1	1	0.90	31	3–90	1.97	0.19–5.71
2002–03	2249	9.9	21	6	9.42	141	56–276	6.27	2.49–12.27
2003–04	2309	7.1	17	7	10.37	108	47–204	4.67	2.03–8.83
2004–05	2424	23.1	21	10	3.74	82	45–132	3.38	1.86–5.45
2005–06	2117	30.6	2	1	0.31	13	2–34	0.60	0.09–1.61
2006–07	2167	28.7	11	5	1.77	55	23–103	2.53	1.06–4.75
2007–08	2164	34.0	20	5	2.72	44	25–74	2.04	1.16–3.42
2008–09	1820	38.1	11	4	1.59	28	13–52	1.55	0.71–2.86
2009–10	2189	30.1	4	2	0.61	30	7–68	1.36	0.32–3.11
2010–11	1551	29.9	7	6	1.51	64	26–116	4.13	1.68–7.48

Annual trawl effort, observer coverage, observed number of common dolphin captures, number of observed capture events, observed capture rate (dolphin per 100 tows), estimated common dolphin captures, and the estimated capture rate (with 95% confidence intervals), in the west coast North Island/New Zealand mackerel trawl fishery.

**Figure 2:**
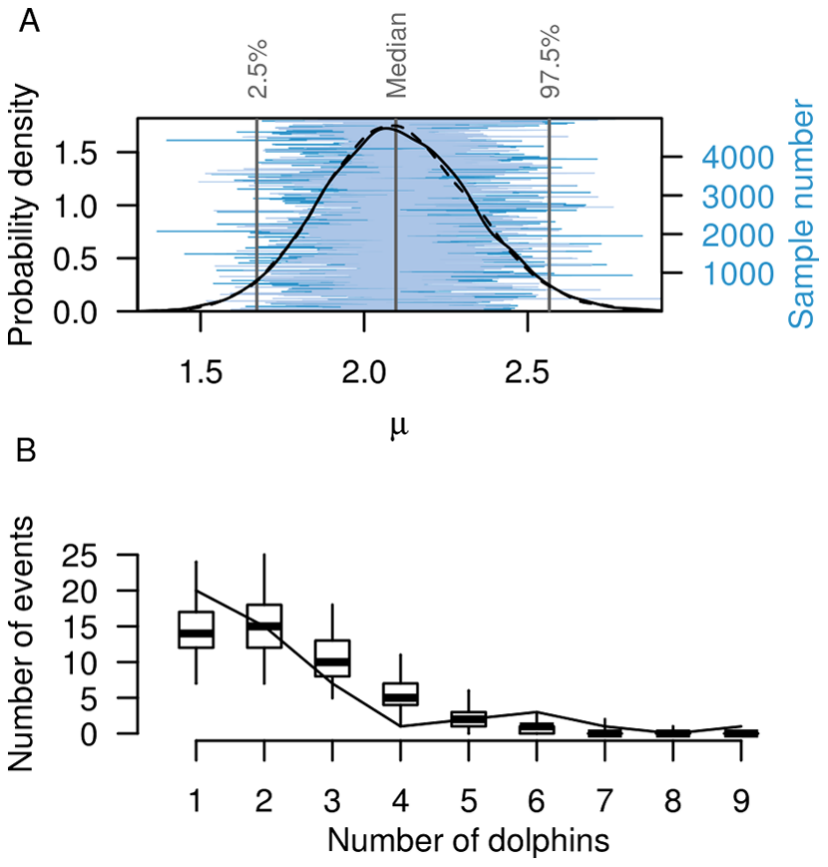
Number of common dolphin *Delphinus delphis* caught per capture event. **A** Posterior distribution of the size of the zero-truncated Poisson distribution, 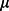, showing the probability density and trace of the two chains. **B** Comparison of the predicted distribution of the number of common dolphin caught per capture event between the observed captures (solid line) and samples from the model posterior (boxplots indicating the median, quartiles, and 95% confidence interval of the distributions).

The 119 incidental captures of common dolphin occurred on 50 of the 2499 observed tows. All captures resulted in mortalities. Typically more than one dolphin was caught per capture event, with two or three dolphins frequently caught at the same time (Figure 2). A maximum of nine individuals was caught in a single incident. There were 0.88 capture events per 100 tows, and an observed capture rate of 2.1 common dolphin per 100 tows across the entire study period.

**Figure 3:**
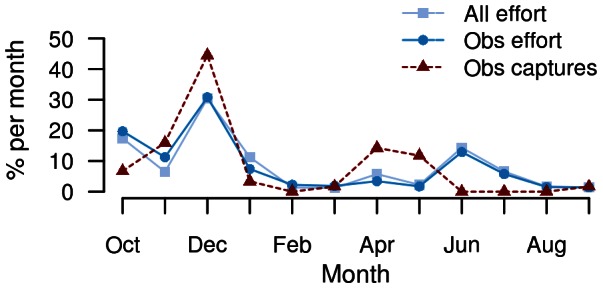
Monthly distribution of fishing effort and common dolphin (*Delphinus delphis*) captures. Total trawl effort, observed effort and observed dolphin captures in the large-vessel mackerel fishery on the North Island's west coast, New Zealand, across the 16-year study period between 1 October 1995 and 30 September 2011.

The spatial distribution of trawl effort in the large-vessel mackerel fishery extended along the North Island west coast, with a similar spatial extent in observer coverage (Figure 1). Both fishing effort and observer coverage were similar in both sub-areas, and observed common dolphin captures occurred in shoreward zones of both sub-areas. Throughout the fishing year, trawl effort was relatively high in October, and showed a marked peak in December, with approximately 20% and 30% of fishing effort occurring in these two months, respectively (Figure 3). Observer coverage corresponded closely with the temporal pattern of fishing effort across months. The number of observed dolphin captures was high in December, and showed another, smaller peak in April and May, at a time when fishing effort was low.

### Estimated common dolphin captures

Based on observer and effort data, the total number of common dolphin captures in the large-vessel mackerel fishery was estimated (Table 2). Over the entire reporting period, the number of estimated common dolphin captures peaked at 141 (95% c.i.: 56 to 276) in 2002–03, and remained relatively high (over 80 estimated common dolphin captures per fishing year) in the two subsequent fishing years. These high estimates were at a time when total fishing effort increased markedly between 1999–00 and 2002–03 from initially low levels. The increase in trawl effort was accompanied by high numbers of estimated common dolphin captures. Since the initial expansion period, the number of tows has generally remained high with over 2000 tows per year, whereas estimated common dolphin captures have fluctuated. In the preceding two fishing years, 2008–09 and 2009–10, there were 28 (95% c.i.: 13 to 52) and 30 (95% c.i.: 7 to 68) estimated common dolphin captures, respectively, with a corresponding annual trawl effort of 1820 and 2189 tows. In the 2010–11 fishing year, there were 64 (95% c.i.: 26 to 116) total estimated common dolphin captures in this fishery. This estimate was the highest value since the 2004–05 fishing year, and higher than estimated common dolphin captures in recent years. It was particularly high considering the concomitant drop in fishing effort in 2010–11 to 1551 tows. Trawl effort in this fishing year was low compared with previous years, and similar to trawl effort in 2001–02, when the fishery was first expanding. The total mean estimated number of captures for the 16-year period was 626 (95% c.i.: 457 to 820).

The high number of estimated common dolphin captures in 2010–11 was reflected in the estimated capture rate of 4.13 (95% c.i.: 1.68 to 7.48) common dolphin per 100 tows. This estimated capture rate was higher than estimated capture rates in the previous six fishing years, and one of the highest estimated capture rates over the entire reporting period.

In addition to predicting the probability of capture events, the two-stage Bayesian model also predicted the number of common dolphin caught per capture event over the 16-year period. This second stage was important, as most capture events involved multiple captures, most frequently two or three common dolphin, with groups of up to nine individuals observed caught at the same time (Figure 2). The posterior distribution of the size of the zero-truncated Poisson distribution, 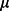, had an approximately normal distribution, with a median value of 2.1 (95% c.i.: 1.7 to 2.6) common dolphin per capture event.

Comparing observer data and model estimates of the number of common dolphin caught per capture event showed that observer data were well represented by the zero-truncated Poisson distribution (Figure 2). All observations were within the 95% confidence intervals of the model estimates, except for the single incident involving the capture of nine dolphins, which was less likely to occur in the model. The 2010–11 fishing year was unusual in that most observed capture events involved individual common dolphin, with only one incident involving the simultaneous capture of two dolphins.

### Model covariates

Selection of potential factors that may explain common dolphin captures confirmed the importance of the four covariates headline depth, tow duration, light condition, and sub-area. Headline depth had the highest explanatory power, followed by tow duration; the other two covariates had considerably less explanatory power. Light condition was included as a three-level factor and, dependent on the time of the haul and the phase of the moon, defined as light, dark, and black light conditions. Based on this analysis, these four covariates were included in the Bayesian model.

**Figure 4:**
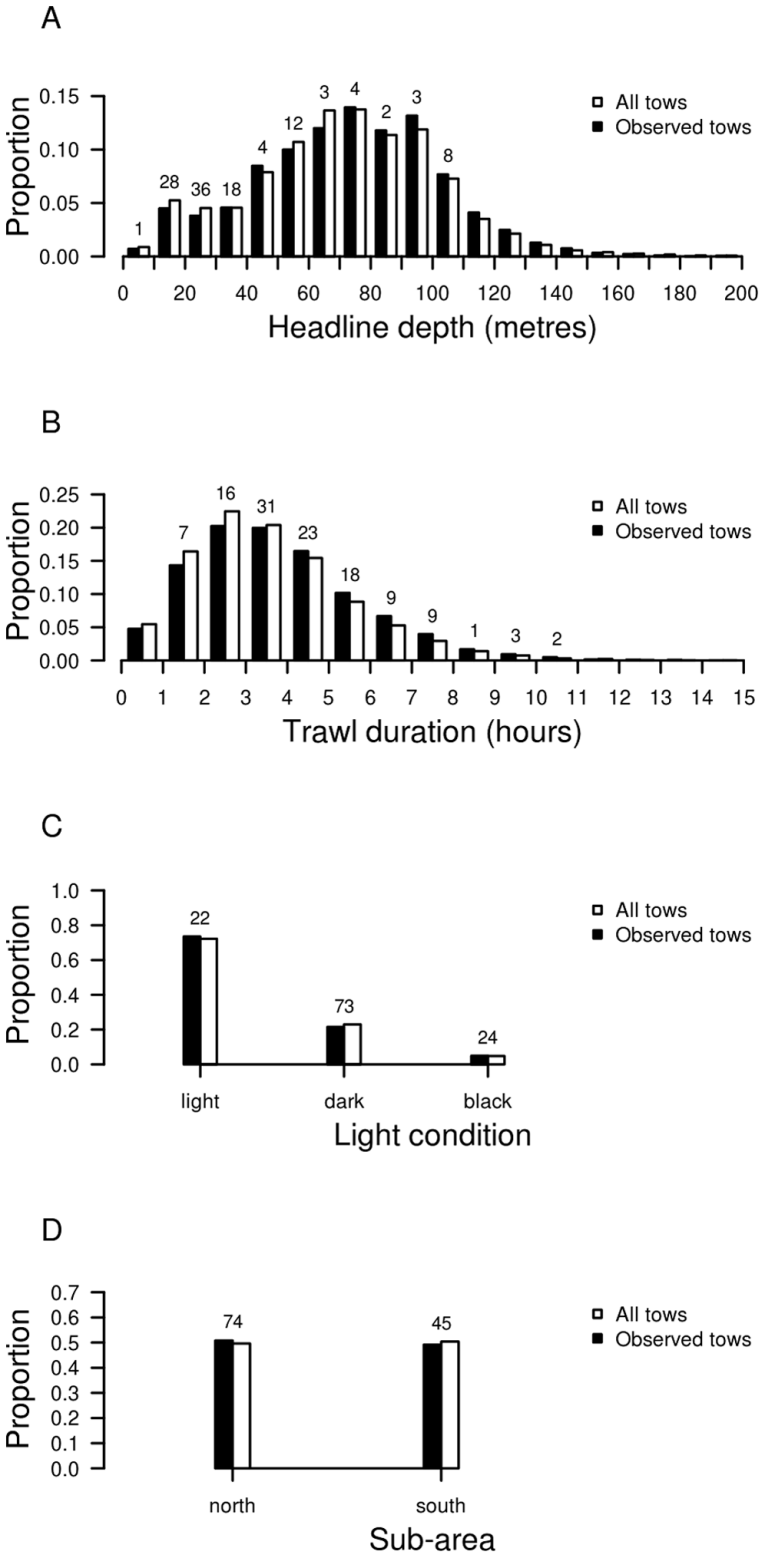
Distribution of selected covariates for the period between 1 October 1995 and 30 September 2011. **A** Headline depth, **B** tow duration, **C** light condition, and **D** sub-area. The covariates were identified as explanatory factors of common dolphin (*Delphinus delphis*) captures in the large-vessel mackerel trawl fishery off the North Island west coast, New Zealand. Total observed common dolphin captures are indicated above the bars.

Comparison of the observed and modelled data sets showed that the distributions of the selected covariates were representative of overall fishing effort (Figure 4). Furthermore, observed common dolphin captures were closely associated with the four covariates. For headline depth, the highest number of observed captures was associated with headline depths between 10 and 40 m, with 83 (70%) of the total 119 observed captures involving tows at headline depths of less than 40 m. There were no observed common dolphin captures at headline depths exceeding 110 m. Tow duration was also an important covariate, and the majority of observed captures (88 captures, 73%) occurred on tows that were between 2 and 6 h in duration. Light condition also influenced common dolphin captures, with dark and black light conditions associated with 95 (80%) observed captures. For the spatial distribution, there was a prevalence of common dolphin captures in the northern sub-area, with 74 (62%) observed captures occurring in this sub-area.

The associated regression coefficients from the model fit were used to quantify the influence of the covariates on the probability of common dolphin captures (Table 3). Headline depth had a negative correlation with a mean coefficient of −0.033 m^−1^, indicating that increasing the headline depth would decrease the probability of a common dolphin capture event. An increase in headline depth by 21 metres would halve this probability. Tow duration was positively correlated with captures, indicating that a decrease in tow duration would decrease the probability of a capture event. Light conditions also influenced the capture event probability, with tows hauled in the light having a mean capture event probability of 0.177 relative to tows hauled in the dark. Tows hauled in black light conditions (i.e., between midnight and dawn on a dark night) had a similar capture event probability (mean 1.078) to tows hauled in the dark.

**Table 3:** Summary of the covariate regression coefficients.

Covariate	Mean	2.5%	50%	97.5%
Headline depth, 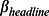	−0.033	−0.045	−0.033	−0.022
Log tow duration, 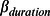	1.470	0.700	1.462	2.285
Light condition, light (relative to dark), 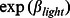	0.177	0.075	0.166	0.346
Light condition, black (relative to dark), 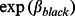	1.078	0.421	1.000	2.139
Sub-area, south (relative to north), 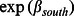	0.539	0.246	0.510	0.996

Covariate regression coefficients presented as mean, and the 2.5%, 50%, and 97.5% quantiles of the posterior distributions. The coefficients of the discrete factors have been exponentiated, so that they are multiplicative, with a value of 1 indicating no effect.

Comparing the two sub-areas, tows in the southern sub-area had about half the capture event probability to those in the northern sub-area, indicated by the mean coefficient of 0.539.

### The mackerel fleet

**Figure 5:**
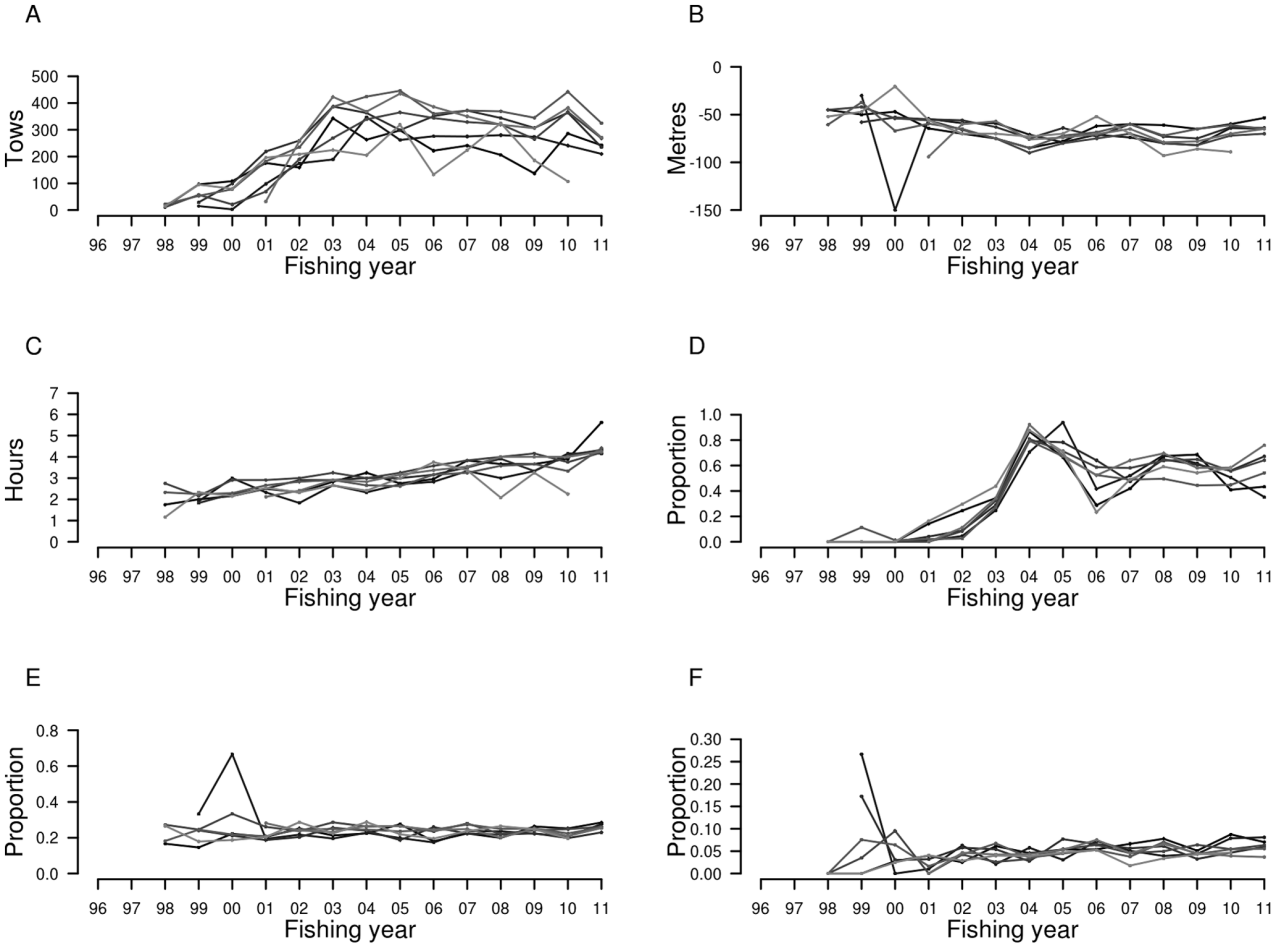
Annual trends of fishing characteristics (covariates) for each of the seven main mackerel trawl vessels. **A** Trawl effort, **B** median headline depth, **C** tow duration, **D** proportion of tows in the north, **E** proportion of tows in dark light conditions, and **F** proportion of tows in black light conditions, for fishing years between 1 October 1995 and 30 September 2011.

There were 15 large vessels operating in the North Island west coast mackerel fishery, with seven vessels accounting for over 95% of the fishing effort in the 16-year period. In general, the mackerel fishery was conducted in a coherent fleet, with main fishing characteristics shared across the seven vessels (Figure 5). Changes in the covariates such as headline depth and light condition occurred at the same time across vessels.

Both trawl effort and tow duration showed an overall increase over the reporting period, with some fluctuations in recent years. Trawl effort declined in 2010–11, following a marked increase the previous year. The decrease in fishing effort in 2010–11 was partly caused by one vessel not participating in this fishery, and also by the remaining vessels fishing less this year. Headline depth showed relatively little variation throughout the study period, and median values have remained below 50 m depth since 2001–02. There was a marked shift to the northern sub-area in 2003–04, with a subsequent return to the southern sub-area in 2005–06. Since 2007–08, the spatial distribution of trawl effort has been relatively even between both sub-areas. Regarding trawl effort in relation to light conditions, the proportions of tows conducted in dark and in black light conditions were also uniform across vessels. Approximately 20% of tows were conducted in dark light conditions, when the net was being hauled between dusk and midnight on a dark night, or between midnight and dawn on a moonlit night. This proportion has remained constant since 2001–02. In comparison, approximately 5% of tows were hauled in black light conditions, with the net being hauled between midnight and dawn on a dark night.

The consistent variation in the covariates across vessels indicated that this fishery was organised into a coherent fleet. Although a specific vessel effect was initially included in the model, it was not significant, and there was no evidence to suggest that particular vessels were better or worse in avoiding common dolphin bycatch.

## Discussion

Between 1995 and 2011, common dolphin captures in the mackerel fishery occurred in most fishing years. All 119 observed captures involved vessels >90 m length, with 15 vessels falling within this category. Nine of these large vessels had an observer on board at least once between 1 October 1995 and 30 September 2011. The two-stage hurdle model, developed to predict the occurrence of any common dolphin capture on a given tow, and the number of dolphin captures per tow (given that there were some captures), fit the data well, and provided plausible estimates when used to predict captures on observed tows.

### Estimated captures

Common dolphin are frequently caught in trawl fisheries worldwide [5], but few studies include sufficient data to estimate capture rates for an entire region or fishery, or to examine temporal trends. As a consequence, previously reported capture rates are generally based on relatively short-term observer data (i.e., 1–2 fishing seasons or <3 fishing years), which are pooled over the entire study period to derive a single capture rate for the fishery. Common dolphin capture rates documented in other trawl fisheries are similar to the higher capture rates reported here. In pelagic trawl fisheries for hake (*Merluccius merluccius*) and sea bass (*Dicentrachus labrax*) in the northeastern Atlantic Ocean, observed capture rates were 7.69 and 10.00 common dolphin per 100 tows, respectively [32]; in the blue whiting (*Micromesistius poutassou*) pair-trawl fishery in the eastern Atlantic Ocean, 8.37 common dolphin were captured per 100 tows [13]. Capture rates in the Atlantic Ocean trawl fishery for large pelagic species (e.g., tuna and swordfish) were 6.0 common dolphin per 100 tows [13].

Over the study period, common dolphin captures in New Zealand waters showed some variation, even though fishing effort has remained relatively high (>1500 tows per year) since 2002–03. The reasons for the fluctuation in estimated common dolphin captures are unknown, but are possibly related to changes in the number of common dolphin in the fishing region. Capture rates are expected to vary from year to year if the dolphin distribution varies significantly over the same period in relation to the fishery [13]. Off the coast of California, United States, common dolphin show significant changes in abundance owing to seasonal and inter-annual shifts in distribution [27]. Common dolphin in New Zealand also show differences in their distribution and habitat use, as they exhibit seasonal inshore-offshore movement, residing in coastal waters during spring and summer, before moving further offshore during autumn [28]. This seasonal migration has been related to changes in sea surface temperature influencing the distribution of their prey species, which in turn may determine the seasonal movement of common dolphin. The distribution of prey has also been implicated in the inshore-offshore migration of common dolphin that occurs on a diel basis, which seems to correspond with the exploitation of different food sources and the movement of prey [25].

As there are no population data available for common dolphin in New Zealand waters [19], and no abundance estimates for this region, it is impossible to establish whether lower estimated capture rates are related to fluctuations in common dolphin abundance at particular fishing locations. Another potential reason for the variation in estimated capture rates could be a change in the vulnerability of this species to being captured in the mackerel fishery. Common dolphin may associate with fishing trawlers to a lesser extent or may become more adept at avoiding captures, as has been proposed for other marine mammals that are able to enter and exit nets without getting caught (e.g., fur seals, [44]).

Fisheries observers in New Zealand record common dolphin sightings, but these data have not yet been collated. Future assessment of observer records of common dolphin sightings may provide clarification whether the decrease in common dolphin captures is related to the number of common dolphin visiting fishing vessels.

Modelling of the entire 16-year data set removed the potential influence of sample size in the present study, but variation in capture estimates in previous studies has been attributed to differences in sample sizes across fishing years, such as the number of tows fished in relation to observer coverage. Substantial deviations in estimated common dolphin capture rates for some years were attributed to small sample sizes rather than actual changes in catch rates in an Atlantic Ocean pelagic pair-trawl fishery [13]. Capture rates in years with limited sampling effort were conspicuously different to those in other years, prompting the pooling of annual data into a single common dolphin capture rate for the study period [13]. Discrepancies between fishing effort and incidental captures of common dolphin were also observed in other trawl fisheries off the United States east coast [33]. Involving foreign vessels from different European countries, these fisheries primarily target squid (*Loligo pealei* and *Illex illecebrosus*) and Atlantic mackerel (*Scomber scombrus*), using off-bottom (high-opening) and pelagic trawls. Assessment of observed incidental captures in relation to fishing effort (days fished) revealed distinct differences in capture rates between countries. For example, Dutch fishing vessels consistently captured up to 10-fold more cetaceans (common dolphin and pilot whale *Globiocephala* spp.) than vessels of the former German Democratic Republic, even though the latter fished a significantly higher number (2–4 times) of days. These differences in capture rates appeared to be related to differences in fishing strategies, such as gear configuration, type of trawl and haulback speed, rather than total fishing effort, but data were insufficient to evaluate this aspect [33].

In the current study, the majority of capture events involved groups of common dolphin, most frequently two to three individuals, with up to nine individuals caught in a single tow. This finding is comparable to observations in other trawl fisheries, which frequently report multiple capture events for this dolphin species. Observer data from different trawl fisheries in the northeast Atlantic Ocean show common dolphin bycatch dominated by groups of two to four individuals [32]. Similarly, pair-trawlers off the Spanish coast mostly caught groups of two to four individuals, with seven and 15 common dolphin involved in one-off multiple capture events [43]. These data confirm that multiple capture events of common dolphin are prevalent in trawl fisheries. Common dolphin frequently form large groups and may also be attracted to fishing vessels, as at-sea survey data of interactions between common dolphin and pelagic pair-trawlers off south-western England suggest [16]. Both the relative abundance of common dolphin and the average group size were greater where trawlers were operating compared with areas where trawlers were absent.

### Headline depth and other covariates

In the present study, detailed data analysis identified the role of four covariates - headline depth, light condition, sub-area and tow duration - in relation to common dolphin captures. The decrease in capture rates in recent fishing years was not associated with a systematic change in any of these covariates. Headline depth (the distance of the headline below the surface) was the covariate that best explained the occurrence of common dolphin captures. Other proxies for tow depth that were considered as covariates were bottom depth, ground-line depth and the height of the net opening, but headline depth had the highest explanatory power. The model estimated that increasing headline depth on a tow by about 21 m would halve the probability of a common dolphin capture occurring. The strong influence of headline depth was also evident in the observer data; 60% of observed capture events, and 70% of common dolphin captured in the large-vessel mackerel fishery occurred on the 14% of observed tows that had a headline depth of less than 40 m. Both the model and observer data suggest that restricting tows with shallow sets would reduce common dolphin bycatch.

Furthermore, across all effort data, 69% of shallow tows (headline depth <40 m) occurred at night. This preference for shallow fishing appears to be related to the prevalence of mackerel in surface waters at night. Diel changes in the behaviour of mackerel have been documented off the coast of Chile [45], where mackerel migrate to the surface during the night and form feeding aggregations. In contrast, they are deeper and more dispersed during the day. Fishing followed this diel movement and headline depth was typically shallower at night coinciding with dark or black light conditions. A similar relationship between common dolphin captures and diurnal movement of prey has been suggested for squid fisheries in the Atlantic Ocean [33]. The upward movement and concentration of squid at the surface seem to concentrate feeding dolphins in surface waters, resulting in the observed higher number of dolphins caught at night (2000-0400h). In addition, if dolphins feed predominantly at night, the likelihood of dolphin captures during the day would be further decreased, as it would spatially separate them from squid during daylight hours [33]. Apart from following the diurnal surface migration of prey, dolphins may also feed and scavenge around trawlers at night-time to reduce competition with other scavenging species such as seabirds [32].

An increased catch of dolphins at night has also been noted in other pelagic trawl fisheries [16], [32], [43], [46]. Furthermore, light was identified as a factor associated with common dolphin captures in a previous analysis of the New Zealand mackerel trawl fishery, although tow depth was also implicated [47]. These studies, however, did not directly investigate the role of headline depth in relation to common dolphin captures, precluding direct comparisons with the findings here. Because of the correlation between headline depth and time of day, the light condition factor only explained a relatively small fraction of the residual deviance. The model showed (Table 3) that the dolphin capture rate was lower for fishing with hauls made in the day or on moonlit nights than at night (a median ratio of 0.17 with a 95% c.i, of 0.08 to 0.35). However, the dolphin capture rate was not significantly different when the haul was between midnight and dawn on dark nights (a median capture rate ratio of 1.00 with a 95% c.i. of 0.42 to 2.14).

### Other fisheries

In addition to incidental captures in pelagic trawl fisheries, common dolphin are also caught in inshore fisheries [48], [49]. For example, in inshore (gillnet and longline) fisheries in northern Peru, common dolphin (predominantly *Delphinus capensis*) were the most commonly observed delphinid captured between 2005 and 2007, constituting 47% of all incidental takes [49]. There have also been reported incidental captures of common dolphin in inshore trawl fisheries in New Zealand [48], but these and other inshore fisheries remain largely unobserved. For example, observer records of inshore trawl fisheries in south-western New Zealand include nine observed common dolphin captures in 2008–09, but these fisheries have not been observed at any other time. Similarly, there has been little (less than 1%) observer coverage of inshore set-net and trawl fisheries operating on the North Island west coast. Inshore fisheries are of concern, as they often overlap with the frequent occurrence of dolphins in shallow coastal waters (e.g., Hector's dolphin *Cephalorhynchus hectori*, [12]; vaquita *Phocoena sinus* [50]). Common dolphin in New Zealand waters have been shown to favour inshore waters during spring and summer, and also during warmer La Niña conditions [28]. The reason for this prevalence appears to be the movement of prey, mainly kahawai (*Arripis trutta*) and jack mackerel, linked to variation in sea surface temperature [28]. In addition, examination of common dolphin diet suggests also diel inshore/offshore movement of this species, including the jack mackerel fishing area [25]. The frequent occurrence of common dolphin in coastal waters makes them vulnerable to inshore fisheries, but scarcity of observer data precludes quantification of incidental captures in this fishing sector.

### Conclusions

Common dolphin are globally distributed, with the population estimated to be in the millions, and a conservation status of “least concern” [21]. Although fishery impacts are not considered a concern at the global population level, they have been found to affect common dolphin populations in particular regions. Common dolphin in the Mediterranean Sea experienced a 50% decline in abundance over three generations, so that their status has recently been listed as “endangered” [51]. In the United States Atlantic Exclusive Economic Zone, fisheries-related mortalities (across all types of fisheries combined) between 1995 and 1999 exceeded the Potential Biological Removal value of this species [52]. The Potential Biological Removal indicates how many individuals can be removed annually without preventing a population (or stock) to reach or maintain its optimal sustainable population levels [53]. A recent assessment of dolphin bycatch on the United States east coast estimated that 14% of the Potential Biological Removal level of this species were captured in the bottom trawl fishery only, with other dolphin species also affected [31].

Common dolphin in New Zealand are considered “not threatened”, even though there are no population estimates for this region [26], [54]. It is unknown whether the west coast North Island population is resident or migratory. While the use of observer data in the present study allowed the development of a statistical model to estimate common dolphin captures in the mackerel fishing region over a 16-year period, the lack of abundance data prevents the assessment of population level impacts of incidental captures in New Zealand waters. In view of the importance of headline depth, it is recommended that nets are towed at headline depths >40 m to reduce the probability of incidental captures of common dolphin.

## Supporting Information

Appendix S1 **BUGS code used for the two-stage Bayesian hurdle model to estimate total common dolphin (** ***Delphinus delphis*** **) captures in New Zealand commercial trawl fisheries between 1995 and 2011.**
(BUG)
